# Twenty years of behavioral nutrition– A reflection on the road less travelled

**DOI:** 10.1186/s12966-025-01804-w

**Published:** 2025-07-25

**Authors:** Nanna Lien, Clare Collins AO, Melanie Hingle, Richard R. Rosenkranz

**Affiliations:** 1 Department of Nutrition, University of Oslo, Oslo, Norway; 2 Nutrition and Dietetics, School of Health Sciences, College of Health, Medicine and Wellbeing, University of Newcastle, NSW, Australia and Hunter Medical Research Institute, Callaghan,, Australia; 3 School of Nutritional Sciences and Wellness, College of Agriculture, Life and Environmental Sciences, University of Arizona, Tucson, USA; 4 Department of Kinesiology and Nutrition Sciences, University of Nevada, Las Vegas, USA

## Abstract

The term behavioral nutrition was introduced at the conception of the International Journal of Behavioral Nutrition and Physical Activity (IJBNPA) twenty-one years ago but it was not formally defined. This commentary describes and contrasts behavioral nutrition against other branches of nutrition sciences such as clinical nutrition and nutritional epidemiology. Examples of how IJBNPA has contributed to the development of behavioral nutrition is provided before concluding with some suggestions for further development. We hope that this will lead to more relevant and innovative submissions of behavioral nutrition research to IJBNPA.

## Introduction

Twenty-one years ago, the first editorial in *International Journal of Behavioral Nutrition and Physical Activity (IJBNPA)* [1] and an accompanying commentary by the founder of the International Society of Behavioral Nutrition and Physical Activity (ISBNPA) [2] introduced the term behavioral nutrition and physical activity, but neither provided specific definitions of either of these terms. Yet physical activity was used consistently, whereas ‘behavioral nutrition’ was used interchangeably with other terms such as diet, nutrition and eating behaviors.

To date, this lack of definition of behavioral nutrition is likely to have contributed to challenges in two areas for *IJBNPA*: (1) A lower number of submissions of manuscripts focused on nutrition (30%) as compared to physical ativity (50%) (unpublished data from the publisher); and (2) Nutrition manuscripts being rejected because they are considered outside the journal’s scope (personal perceptions by *IJBNPA* editors).

The aims of this commentary are to describe the nutritional and behavioral components of behavioral nutrition in a historical context, provide relevant examples of *IJBNPA* articles which have furthered the understanding of behavioral nutrition, and suggest future areas for research on behavioral nutrition.

### Emergence of the terms nutritional and behavioral

There is a lack of clarity in the definition of what constitutes nutrition science, but there is agreement that modern nutrition science developed from chemical and biological sciences with the discovery of vitamins and research on micronutrient deficiencies conducted in the early 20th century [3, 4]. This foundation of nutrient deficiency research led to formation of the American Society for Nutrition and publication of the first nutrition journal in 1928 which was followed by three more nutrition journals. In the United Kingdom, the Nutrition Society was established in 1941 and currently has six scientific journals. The Nutrition Society was also instrumental in establishing two umbrella organizations: The International Union of Nutritional Sciences (IUNS) in 1948 and the Federation of European Nutrition Societies (FENS) in 1973. Through their conferences, they helped shape the field of nutrition science both in Europe and globally. However, subject-specific fields of nutrition also emerged as described below.

In the US in the 1950s, the merging of nutrition with medical research led to establishment of the field of clinical nutrition with support from what is now the American Nutrition Association (ANA). While ANA has moved on to personalized nutrition [5], the European Society for Clinical Nutrition and Metabolism (ESPEN) recently stated that “any nutritional measure, preventive or curative, targeting individual patients is clinical nutrition” [6]. The field of clinical nutrition thus overlaps with the dietetics profession and their association established in the US in 1917, but with the dietetic skills now being applied internationally in many settings other than healthcare [7]. In the patient setting, behavioral nutrition would focus on how to change dietary intake behaviors and on strategies used to support adherence to the dietary advice, not on the effects on clinical outcomes of the patients.

Nutritional epidemiology was established as a subfield of epidemiology in the 1980s and addresses the aspects of diet that may influence the development of human disease [8]. Methods for assessing dietary intake are a key aspect of nutritional epidemiology, for which food composition data are needed to calculate macro- and micronutrient intakes [8]. This has also informed development of the International Conference on Diet and Physical Activity Methods (ICDAM) which has informed reviews and recommendations on dietary assessment methods [9, 10]. In behavioral nutrition, comprehensive dietary assessment is not commonly used as the focus is often on specific dietary behaviors. When epidemiologists investigate factors that influence the distribution of diseases in the population, their main focus is on risk factors such as diet and the variation by socio-demographic factors. However, in behavioral nutrition, a multitude of factors at individual, interpersonal, community and societal levels are studied [11–13] not only to understand the distribution of risk of diseases in the population, but also to promote health and well-being through dietary behaviors.

Since the 1950s, nutrition knowledge has been applied to agricultural and food sciences to address global hunger through increased food production and food processing to extend shelf life and reduce post-harvest waste. Concurrently, nutrition education was applied to translate the knowledge gained from nutrition science into population recommendations in order to optimize food patterns and nutrient intakes for prevention of deficiency diseases. In the United States, the Society for Nutrition Education was important for advancing this translation but did not become the “Society for Nutrition Education and Behavior” until 2012. In the 1980s and 1990s the behavioral focus was further strengthened by research in health education and health promotion that focused on promoting healthy dietary patterns [14]. The original narrow biological and reductionist focus on nutrition science was challenged in the late 1990s and early 2000s as the prevalence of diet-related non-communicable diseases (NCDs) rose, prompting a number of activities, including the creation of the journal Public Health Nutrition; the Giessen Declaration on the new nutrition science moving beyond the reductionist paradigm [15]; and the World Public Health Nutrition Association. This aligned well with the concurrent development of the concept of behavioral nutrition within ISBNPA.

Skinner [16] defined behavior as any observable and measurable activity of an organism and further emphasized that behavior is a function of environmental events and can be analyzed scientifically [17]. Applying this to nutrition indicates that our interest is understanding the why, how, where, when, and under what conditions people eat, and less about the relationship between nutrients and health outcomes or socio-demographic variation in terms of foods consumed or dietary patterns. Within the field of psychology, the terms ‘eating behaviors’, ‘eating practices’ or ‘eating habits’ have been used to describe food behavior [18]. Other terms used are ‘dietary behaviors’ [19] which might be preferred by those using food-based dietary guidelines (FBDG) to implement population-level recommendations [20], and ‘food choices’ [21] which might be more common within sociology and consumer sciences. Regardless of the term used, the aim is to understand the stimulation, activation or promotion of behaviors related to a nutrition outcome. This is to further support adoption of behaviors that help the individual or population align food consumption patterns with FBDGs, while ameliorating those that do not, such that NCDs are prevented or delayed, and quality of life, health and well-being is increased. In the 1990s and early 2000s, personal factors influencing behaviors were the focus of both consumer sciences and psychology drawing on theories such as Theory of Planned Behavior and Social Cognitive Theory among others [14]. A recent model drawing on such behavioral theories is the COM-B where behavior is seen as a result of personal motivation and capability, as well as opportunity [22]. From the early 2000s, there has been an increasing research focus on both the proximal and distal social and physical environments on the individual behavior, guided by the Social Ecological Model [23]. This is in line with Skinner’s point that behavior might be shaped by environmental stimulation and the recent argument by Olstad and Kirkpatrick [24] that dietary behavior could better be named ‘eating practices and patterns’ to avoid an overly simplistic focus on individual choice.

### The contribution of *IJBNPA* to behavioral nutrition

From its inception, the editors of *IJBNPA* have emphasized the importance of behavioral processes in preventing obesity and diet-related NCDs and improving overall well-being. The editors have prioritized innovative, interdisciplinary research across levels of influence [20] and incorporated diverse methodologies and theoretical approaches to address the complex determinants of behavioral nutrition worldwide. All these characteristics have remained fixtures of *IJBNPA*, including an emphasis on developing and evaluating models to better understand, and influence, health-related behaviors in diverse cultural contexts.

Investigations of predictors or correlates of behavioral nutrition include: Psychological– such as impulsivity [25] and motivation [26]; Social environment– such as parenting practices [27, 28] and cultural factors [29]; as well as the Physical environment– including both accessibility to food outlets and availability of foods and cues within these, as well as pricing and marketing aspects [30–32]. Furthermore, newer methods to investigate the influences and interactions of these determinants have included mediation [33, 34], moderation [35] and multilevel analysis [36]. Interventions targeting determinants of behavioral nutrition have been tested in (group) randomized controlled trials (RCTs) conducted in clinical practice [37], the community [38], schools [39] and child care [40], as well as home-based programs for seniors [41]. Some behavioral interventions have also been conducted in virtual [42, 43] or real [44] supermarkets, and the possibilities of new technologies in delivering interventions have been explored [45–47]. The interest in understanding the strategies, determinants and outcomes of implementation of the behavioral nutrition interventions has increased [48–50] as the effects of such interventions have often been small at best. Recently, participatory and co-creation approaches have been applied in the development of the interventions to address the small effects [51].

Research on innovative behavioral theories includes the development and testing of new or adapted theories that explain and predict behavioral nutrition [52–56]. Measurement and methodological challenges include research that seeks to improve the tools and methods used to assess both dietary intake and behavioral nutrition aspects in near real time through use of technology [57–60], as well as measurement of determinants such as parental practices [61], food environment [62, 63] or marketing to children [64]. Examination of how policy interventions can promote healthier dietary behaviors at the population level was a focus already early on through research on marketing, labeling and portion sizes [65–67],and this has increased in recent years with the understanding that obesity and NCDs are wicked problems that need to be addressed by national-level policies [68–70] and applying a systems lens [71–73] to understand and change behavioral nutrition.

### The future of behavioral nutrition

Advancing behavioral nutrition relies heavily on transdisciplinary research, drawing from fields such as epidemiology, psychology, sociology, environmental and ecological sciences, technology, political science, economics, implementation science, and system science to build a more comprehensive understanding of what influences behavioral nutrition and how to effectively promote change.

An early perspective on behavioral nutrition underscored the fundamental idea that nutrition goes beyond physical health to significantly affect mental and psychological states [74], setting the stage for modern explorations into how nutrition influences mood, cognitive functions, and overall behavior. Future research on the connection between behavioral nutrition, mental health, and cognitive function is likely to address several emerging and complex questions, focusing on the interplay between dietary patterns, metabolites, psychological well-being, prevention, risk reduction and management of diseases (such as Parkinson’s, Alzheimer’s, chronic traumatic encephalopathy, traumatic brain injury) and cognitive performance among students, workers, and adults as they age [54, 75–77]. Mental health problems, as well as obesity, are major public health problems globally and the possible connection between the two through behavioral nutrition is worth exploring further according to the youth in the CO-CREATE project [78].

Precision and personalized nutrition utilize knowledge from an individual’s genotype, phenotype, health status, dietary intake, and other clinical data including the metabolome and microbiome, to develop individually tailored nutrition recommendations [79]. In the future, these data may also be used to help explain individual variation in behaviors and treatment response and potentially be used within routine clinical care to guide timely treatment decisions.

The interest in potential use of artificial intelligence (AI) is rapidly increasing and is likely to have an influence on behavioral nutrition research moving forward. Currently, AI is demonstrating utility in generating basic nutrition advice [80], but its accuracy is reduced in complex health conditions [81] and at this stage not able to replicate health professional interventions. Yet, there are some promising results using chatbots in digital health interventions to promote healthier beverages [82]. Furthermore, AI has the potential to reduce both cost and burden of dietary behavior monitoring, but the issues of algorithm validity and reproducibility will need to be addressed [83]. Current limitations and heterogeneity within research conducted to date mean that further research is required to evaluate the potential of AI within behavioral nutrition [84].

The United Nations 2030 Agenda for Sustainable Development includes 17 goals, which collectively frame the global actions and innovative research needed to end hunger and prevent diet-related NCDs and obesity, as well as ensure the sustainability of our planet. This agenda has led to calls for greater use of behavioral sciences in public health [85] and transdisciplinary research in nutrition [86], as well as for applying a complex adaptive system lens on human behavior [87] and an increased focus on the commercial determinants resulting from the activities of the private sector [88]. Thus, the broad concept of nutrition as defined in the Giessen Declaration on the new nutrition science [4, 15] and practiced in IJBNPA is as relevant as ever.

## Conclusion

Despite the lack of agreement on a single definition of nutrition sciences, the history of its development can be traced from the biochemical origin branching into clinical, epidemiological, and public health nutrition. The latter coincided with an increased focus of the behavioral aspects of nutrition in the late 1990s and early 2000s, and with the founding of ISBNPA and *IJBNPA*. *IJBNPA* has clearly contributed to driving the research on behavioral nutrition through furthering the understanding of determinants and behavior change aspects of preventing NCDs and obesity, as called for in the journal’s first commentary [2]. The current momentum to solve nutrition-related physical and mental health problems, as well as sustainability challenges through behavioral research across population, systems and personalized perspectives with or without AI will ensure that *IJBNPA* will continue to develop behavioral nutrition. We look forward to witnessing the next 20 years of behavioral nutrition research as it continues to develop and address crucial issues for populations across the globe.

## Data Availability

No datasets were generated or analysed during the current study.

## Abbreviations

AI: Artificial intelligence
ESPEN: European society for clinical nutrition and metabolism
FBDG: Food-based dietary guidelines
FENS: Federation of European Nutrition Societies
ICDAM: International Conference on Diet and Physical Activity Methods
IJBNPA: International Journal of Behavioral Nutrition and Physical Activity
ISBNPA: International Society of Behavioral Nutrition and Physical Activity
IUNS: International Union of Nutritional Sciences
NCD: Non-communicable disease
